# Time evolution of cytokine profiles associated with mortality in COVID-19 hospitalized patients

**DOI:** 10.3389/fimmu.2022.946730

**Published:** 2022-09-27

**Authors:** Laura Sánchez-de Prada, Óscar Gorgojo-Galindo, Inmaculada Fierro, Ana María Martínez-García, Guillermo Sarmentero-López de Quintana, Rocío Gutiérrez-Bustillo, María Teresa Pelaez-Jareño, Elisa Álvarez-Fuente, Esther Gómez-Sánchez, Eduardo Tamayo, Álvaro Tamayo-Velasco, Marta Martín-Fernández

**Affiliations:** ^1^ BioCritic, Group for Biomedical Research in Critical Care Medicine, Valladolid, Spain; ^2^ Microbiology and Immunology Department, Hospital Clínico Universitario de Valladolid, Valladolid, Spain; ^3^ Department of Surgery, Faculty of Medicine, Universidad de Valladolid, Valladolid, Spain; ^4^ Faculty of Health Science, Universidad Europea Miguel de Cervantes, Valladolid, Spain; ^5^ Anesthesiology and Critical Care Department, Hospital Clínico Universitario de Valladolid, Valladolid, Spain; ^6^ Centro de Investigación Biomédica en Red de Enfermedades Infecciosas (CIBERINFEC), Instituto de Salud Carlos III, Madrid, Spain; ^7^ Haematology and Hemotherapy Department, Hospital Clínico Universitario de Valladolid, Valladolid, Spain; ^8^ Department of Medicine, Faculty of Medicine, Universidad de Valladolid, Valladolid, Spain

**Keywords:** COVID-19, cytokines, mortality, principal component analysis - PCA, hospitalized patients

## Abstract

**Background:**

High cytokine levels have been associated with severe COVID-19 disease. Although many cytokine studies have been performed, not many of them include combinatorial analysis of cytokine profiles through time. In this study we investigate the association of certain cytokine profiles and its evolution, and mortality in SARS-CoV2 infection in hospitalized patients.

**Methods:**

Serum concentration of 45 cytokines was determined in 28 controls at day of admission and in 108 patients with COVID-19 disease at first, third and sixth day of admission. A principal component analysis (PCA) was performed to characterize cytokine profiles through time associated with mortality and survival in hospitalized patients.

**Results:**

At day of admission non-survivors present significantly higher levels of IL-1α and VEGFA (PC3) but not through follow up. However, the combination of HGF, MCP-1, IL-18, eotaxine, and SCF (PC2) are significantly higher in non-survivors at all three time-points presenting an increased trend in this group through time. On the other hand, BDNF, IL-12 and IL-15 (PC1) are significantly reduced in non-survivors at all time points with a decreasing trend through time, though a protective factor. The combined mortality prediction accuracy of PC3 at day 1 and PC1 and PC2 at day 6 is 89.00% (p<0.001).

**Conclusions:**

Hypercytokinemia is a hallmark of COVID-19 but relevant differences between survivors and non-survivors can be early observed. Combinatorial analysis of serum cytokines and chemokines can contribute to mortality risk assessment and optimize therapeutic strategies. Three clusters of cytokines have been identified as independent markers or risk factors of COVID mortality.

## Introduction

Coronavirus disease 2019 (COVID-19) has emerged as a global infectious respiratory disease caused by a novel betacoronavirus: the SARS- CoV-2 (severe acute respiratory syndrome coronavirus 2) (1, 2). Since its emergence in December 2019 until December 2021, it has infected nearly 300 million people and killed more than 5 million people across the world (3). COVID-19 infection displays a wide clinical presentation ranging from asymptomatic or mild symptoms to severe pneumonia and critical respiratory failure (4). Due to its complex physiopathology, prognosis at hospital admission remains a challenge.

Severe COVID-19 has been associated with an acute hyperimmune response known as “cytokine storm” or “cytokine release syndrome” (CRS) contributing to multiorgan failure (5). Recent studies suggest its relation to an impaired type I IFN immunity (6, 7). That hypercytokinemia, has been correlated with respiratory failure, ARDS and adverse clinical outcomes (8–10). Cytokines released by immune cells in case of uncontrolled inflammation, have been studied as markers and profiles of severity to predict outcomes (11–16). Although individual cytokine analysis can shed some light on COVID-19 pathology, the immune responses are complex, and a combinatorial analysis could better explain the relations between cytokine levels. Previous studies with principal component analysis (PCA) and transcriptomic have determined the cytokine profiles regarding COVID-19 severity and mortality (7, 17–19). However, differences between the different cytokine profiles and how they evolve through time in hospitalized patients, comparing mortality and survival has not been performed yet.

In this study, we aimed to establish the timeline of different cytokine profiles that help to predict patient outcome in order to anticipate, adapt treatment and improve survival.

## Materials and methods

### Study design

We performed a prospective study at Hospital Clínico of Valladolid (Spain). A total of 108 patients with RT-PCR-confirmed SARS-CoV2 infection and were recruited from late March to 11th April of 2020. Patients with other active infections or a terminal chronic disease were excluded. Patients were divided into two groups according to the occurrence of mortality. In addition, 28 age and gender matched healthy volunteers with a negative RT-PCR test for SARS-CoV2 infection were also recruited during routine pre-anesthetic evaluation for scheduled surgery. The present study was approved by the Valladolid Hospital’s Clinical Ethics Committee (CEIm) (cod: PI 20-1717) and all subjects provided a written informed consent.

Plasma samples were collected the first, third and sixth day of hospital admission in 3.2% sodium citrate tubes and centrifuged at 2000 g for 20 minutes at room temperature. Plasma was aliquoted and stored at -80°C until used.

### Cytokine analysis

Concentration of 45 cytokines in plasma was determined by 45-plex Human XL Cytokine Luminex Performance Panel (R&D), following manufacturer instructions. The cytokines analyzed are the following ones: BDNF, EGF, eotaxin (also known as CCL11), FGF-2, GM-CSF, GRO-α (CXCL1), HGF, IFN-α, IFN-γ, IL-1α, IL-1 β, IL-10, IL-12 p70, IL-13, IL-15, IL-17a (CTLA-18), IL-18, IL-1RA, IL-2, IL-21, IL-22, IL-23, IL-27, IL-31, IL-4, IL-5, IL-6, IL-7, IL-8 (also known as CXCL8), IL-9, IP-1 beta (CCL4), IP-10 (CXCL10), LIF, MCP-1 (CCL2), MIP-1α (CCL3), NGF-β, PDGF-BB, PIGF-1, RANTES (CCL5), SCF, SDF-1α, TNF-α, TNF- β, VEGF-A, and VEGF-D.

### Statistical analysis

Since most of the cytokine data did not follow a normal distribution, continuous variables were presented in terms of median (interquartile range, IQR) and compared among groups using the Mann-Whitney U-test or T-test. Categorical variables were described as count and percentages, which were compared using the χ2 test or Fisher exact test, when appropriate.

Distributions of the cytokine values were assessed and log2 transformed to render the principal component analysis (PCA). PCA was carried out with all variables related to cytokine concentrations that showed significant differences between survivors and non-survivors in the univariate analysis, on at least one of the three analyzed days. All participants for whom the variables of interest were available were included in the final analysis and no assumptions were made for missing data. Prior to extraction of factors, Kaiser-Meyer-Olkin (KMO) measure of sampling adequacy and the Bartlett test of sphericity were checked to evaluate the fitness of the data for factor analysis. The factor solution was formed based on the eigenvalues, which represent the amount of variance captured by given components. Factors with eigenvalues >1.0 were retained, according to the Kaiser-Guttman criterion and the Scree Plot. Then we optimized the factor solution using varimax rotation. A factor-based-score was calculated for each component, and we estimated the factor scores on all COVID-19 patients at the first, third and sixth day of admission.

To assess changes over time in outcome measures within each group, Friedman’s repeated measures test with Dunn’s multiple comparison test was used for non-parametric data and a repeated-measures analysis of variance (RM-ANOVA) with Tukey’s multiple comparison test for parametric data.

A multivariable logistic regression analysis was performed to estimate the relevance of each factor obtained in the PCA analysis, differentiating the profile of the patients who survived versus those who did not survive 28 days after admission. The logistic regression model was internally validated by performing bootstrap resampling (1000 resamples). The area under the receiver operating characteristic curves (AUC ROC) shows the accuracy for the final logistic regression model and individual predictors. All statistical analyses were performed using SPSS version 28.0 for Windows (SPSS, Inc, Chicago, Ill). A p-value of less than 0.05 was regarded as statistically significant.

## Results

### Characteristics of the patients

A total of 108 hospitalized subjects admitted with clinical SARS-CoV-2 pneumonia were enrolled, of whom 20 died within 28 days after admission and composed the non-survivors’ group. The other 88 patients were included in the survivors’ group. Baseline characteristics of patients are reported in **Table 1**. The group of non-survivors was significantly older than the other group. There were no differences in terms of gender and comorbidities between groups. Considering the analytical variables, non-survivors presented higher levels of glycaemia, creatinine, leukocytes, neutrophils, procalcitonin, CRP, D-dimer and LDH. There were no significant differences in clinical outcomes referring to the percentage of patients with invasive mechanical ventilation and length of stay in hospital and intensive care unit.

**Table 1:** Clinical characteristics of the patients. Data are represented as [median (IQR)] and as [% (n)].

	Non-survivors at 28 days(N=20)	Survivors(N=88)	p-value
Age in years [median (IQR)]	73.5 (14)	67 (17)	0.017
Male [n (%)]	12 (60)	47 (53.4)	0.593
**Comorbidities [n (%)]**			
Smoking	4 (20)	5 (5.7)	0.059
Coronary disease	2 (10)	8 (9.1)	1.000
Atrial fibrillation	4 (20)	8 (9.1)	0.229
Diabetes	5 (25)	14 (15.9)	0.340
Neurological disease	1 (5)	1 (1.1)	0.337
Stroke	0 (0)	1 (1.1)	1.000
Hypertension	11 (55)	39 (44.3)	0.460
Liver disease	1 (5)	1 (1.1)	0.337
Obesity	2 (10)	8 (9.1)	1.000
COPD	2 (10)	5 (5.7)	0.611
Kidney disease	2 (10)	1 (1.1)	0.087
**Laboratory. [median (IQR)]**			
Glycaemia (mg/dL)	198 (227)	106 (67.25)	<0.001
Creatinine (mg/dL)	0.995 (0.86)	0.815 (0.23)	0.007
Total bilirubin (mg/dL)	0.5 (0.58)	0.5 (0.39)	0.482
Leukocytes (x10^9^/L)	8.16 (10.23)	6.41 (3.72)	0.042
Lymphocytes (x10^9^/L)	0.72 (0.74)	1 (0.56)	0.185
Neutrophil (x10^9^/L)	7125 (9590)	4725 (3272.5)	0.016
Procalcitonin (ng/ml)	0.3 (0.57)	0.09 (0.195)	<0.001
Platelet (x10^9^/L)	195 (95.25)	208 (117)	0.512
CRP (mg/L)	160 (190)	76 (95.5)	0.003
Ferritin (µg/L)	1024.5 (113.25)	671 (1107)	0.126
D-dimer (mg/L)	2029 (23629.25)	711 (769.5)	0.015
LDH (mmol/L)	385 (183.25)	306 (96.25)	0.002
**Clinical outcomes**			
Invasive mechanical ventilation [n (%)]	12 (60)	21 (23.9)	0.122
Length of hospital stay [days.median (IQR)]	15.5 (11.75)	11 (13.5)	0.153
Length of ICU stay [days. median (IQR)]	17.5 (8.75)	20 (19.5)	0.253

IQR, interquartile range; COPD, chronic obstructive pulmonary disease; CRP, C-Reactive protein; ICU, intensive care unit.

### Cytokine profile

In bivariate analysis, the levels of 10 cytokines were different (p< 0.05), at least in one of the three moments (1, 3 and 6 days) between survivors and non-survivors (**Tables S1-3**). On the first day of admission (**Table S1**), significant differences were observed regarding HGF, IL-2, IL-1α, IL-15 and VEGFA levels. IL-15 and IL-2 were the only cytokines whose levels were significantly reduced by half in the group of non-survivors at 28 days. The rest of cytokines were significantly increased in this group. Specifically, IL-1α and VEGFA quadrupled and doubled their levels, respectively. On the third day of admission (**Table S2**), differences between groups were found in the case of BDNF, eotaxin, IL-18 and again HGF and IL-15 levels. In non-survivors, levels of HGF and IL-18 were significantly higher, where HGF tripled the levels compared to survivors. On the other hand, levels of BDNF, eotaxin and IL-15 were significantly lower in this group. Finally, on the sixth day of admission (**Table S3**), cytokines significantly elevated in non-survivors were SCF, MCP-1, IL-18, VEGFA and HGF. The exception became again IL-15, which barely showed variation over time, and whose levels in non-survivors remained the half of those in survivors. IL-18 increased its levels compared to day 3 of admission; and VEGFA, which significantly doubled the level on the first day of admission, tripled the levels in non-survivors on the sixth day. However, HGF stands out as the only cytokine that remained significantly elevated in the group of non-survivors through all three measurement times, reaching its maximum peak and difference compared to survivors, on the sixth day of admission.

Some of these 10 cytokines and chemokines (IL-1a, IL-2, IL-15, IL-18, eotaxin, HGF, MCP-1, SCF and VEGFA) have been linked to different aspects of COVID-19 disease, its severity and even mortality but a combinatorial analysis can help us to understand its complex interaction. Principal component analysis (PCA) is a technique for reducing the dimensionality of such datasets, increasing interpretability but at the same time minimizing information loss.

### Principal component analysis

The Kaiser-Meyer-Olkin measure of sampling adequacy (0.7) and the Bartlett test of sphericity (p < 0.001) indicated that the factor matrix was adequate for data (**Table S4**).

**Figure 1:**
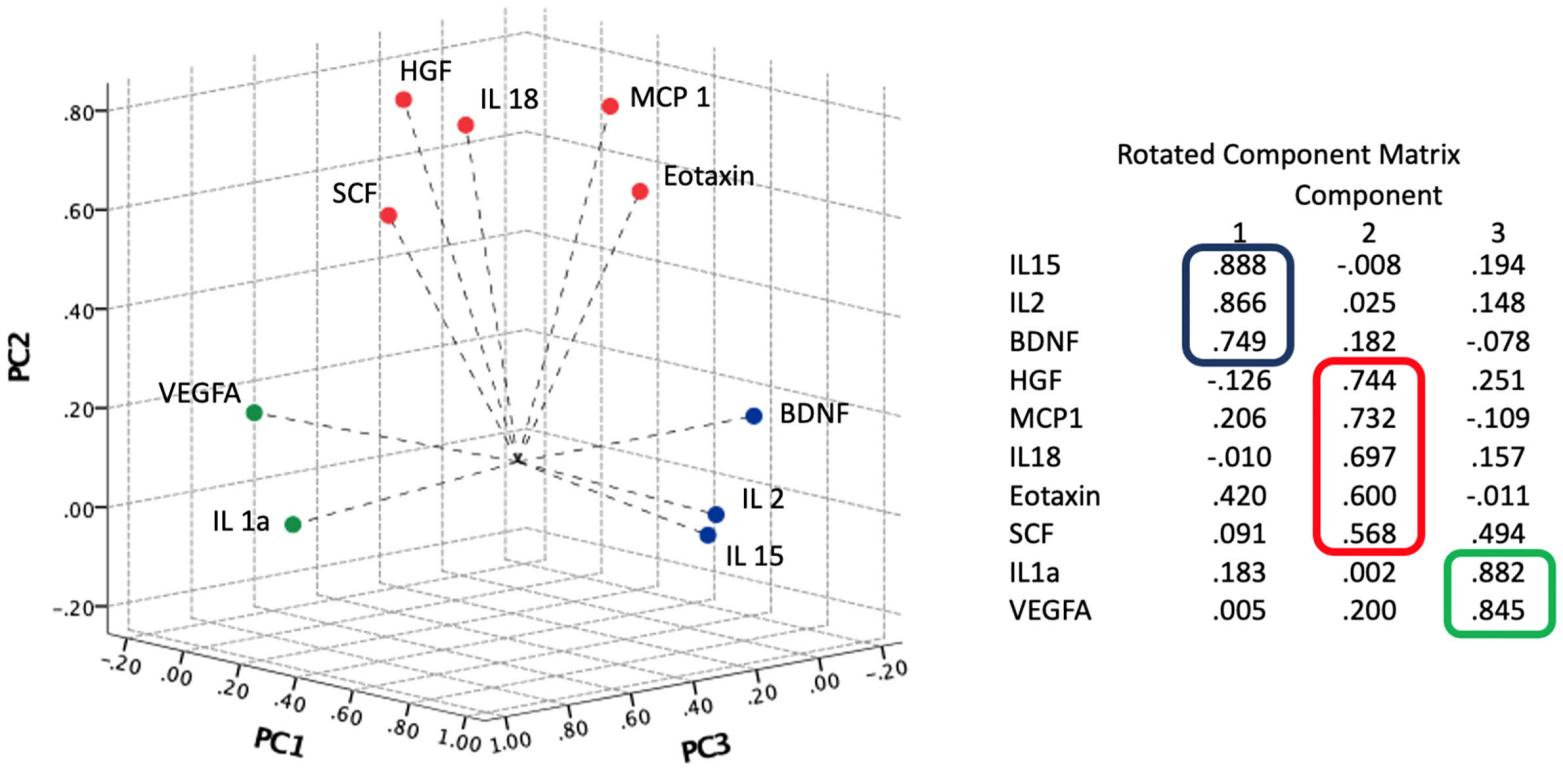
Component plot in rotated space. Results of principal component analysis (PCA) and Varimax rotation method with Kaiser normalization. The three components accounted for 66.09% of the total variance.

PCA resulted in three components with eigenvalues greater than one that describe relationships between cytokines’ levels in patients with COVID-19. The three components accounted for 66.09% of the total variance. PC1, PC2, and PC3 accounted for 32.63%, 19.14%, and 14.32% of the variance, respectively. PC1 is composed by IL-15, IL-2 and BDNF; PC2 by HGF, MCP-1, IL-18, eotaxin and SCF; and PC3 by IL-1α and VEGFA. **Figure 1** show three-dimensional score plot illustrating how cytokines were distributed on PCs.

The algorithms of the principal components were applied to all patients and calculated scores were saved as new variables PC1, PC2 and PC3 at the first, third and sixth day of admission.

### Principal components score in COVID patients vs control group

In addition, the algorithms of the principal components were applied to the control group and the PCA scores (PC1, PC2 y PC3) were compared between COVID-19 and healthy SARS-CoV-2-negative control subjects on the day of admission. The calculated scores were higher in COVID-19 patients than in controls: PC1 (t_(52.26)_= 3.53; p=0.001); PC2 (U= 988.00; p= 0.005); PC3 (U= 1111.00; p= 0.031). These values let us confirm that those cytokines are different between infected and healthy subjects, emphasizing the importance of these cytokines in COVID-19 infection.

### Principal components score in survivors *vs* non-survivors

**Figure 2:**
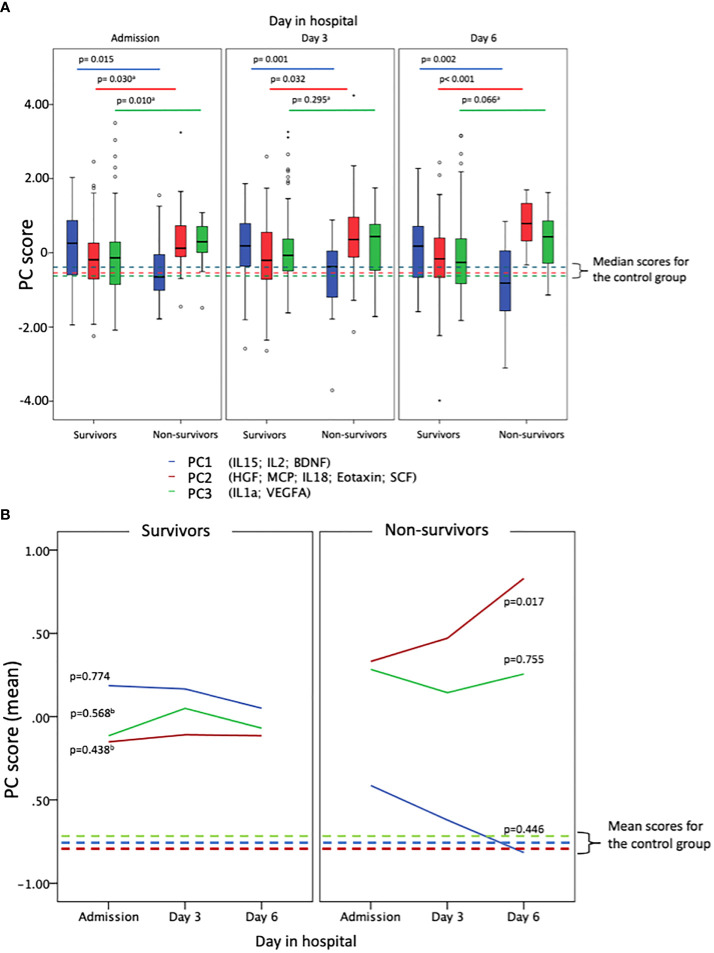
PC scores for COVID patients (survivors and non-survivors) and controls when applying PC estimators. In blue PC1 groups IL-15; IL-2 and BDNF. In red PC2 groups HGF; MCP1; IL-18; eotaxin and SCF. In green PC3 groups IL-1a and VEGFA. **(A)** Box-plots shows the different contribution of each of the factors obtained in the analysis of principal components for survivors and non-survivors, showing, in addition, three moments in the evolution of the disease (first, third and sixth days of admission of the patients). **(B)** Shows that in the first 6 days of hospitalization, the mean score is stable and significantly lower for patients who survived than for those who died. In addition, a growing trend is observed for this marker in patients who did not survive. Trends over time of the PC scores for survivors and non-survivors were calculated separately by using RM-ANOVA or Friedman test.

**Figure 2** shows PC1, PC2 and PC3 scores at the first, third and sixth day of admission for survivors and non-survivors. In **Figure 2A**, significant differences are observed between both groups. Mortality was associated with lower scores of PC1 and higher scores of PC2 and PC3. This indicates that higher scores in PC1 are protective while PC2 and PC3 represent risk factors. In contrast to PC3, which is only significantly elevated in non-survivors at day one; the differences between survivors and non-survivors in the cytokines of PC1 and PC2 intensified through time. **Figure 2B** shows that in the first 6 days of hospitalization, the mean score is stable and significantly lower for patients who survived than for those who died. In addition, a growing trend is observed for this marker in patients who did not survive.

### Multivariate analysis of principal components associated with mortality at 28 days

**Figure 3:**
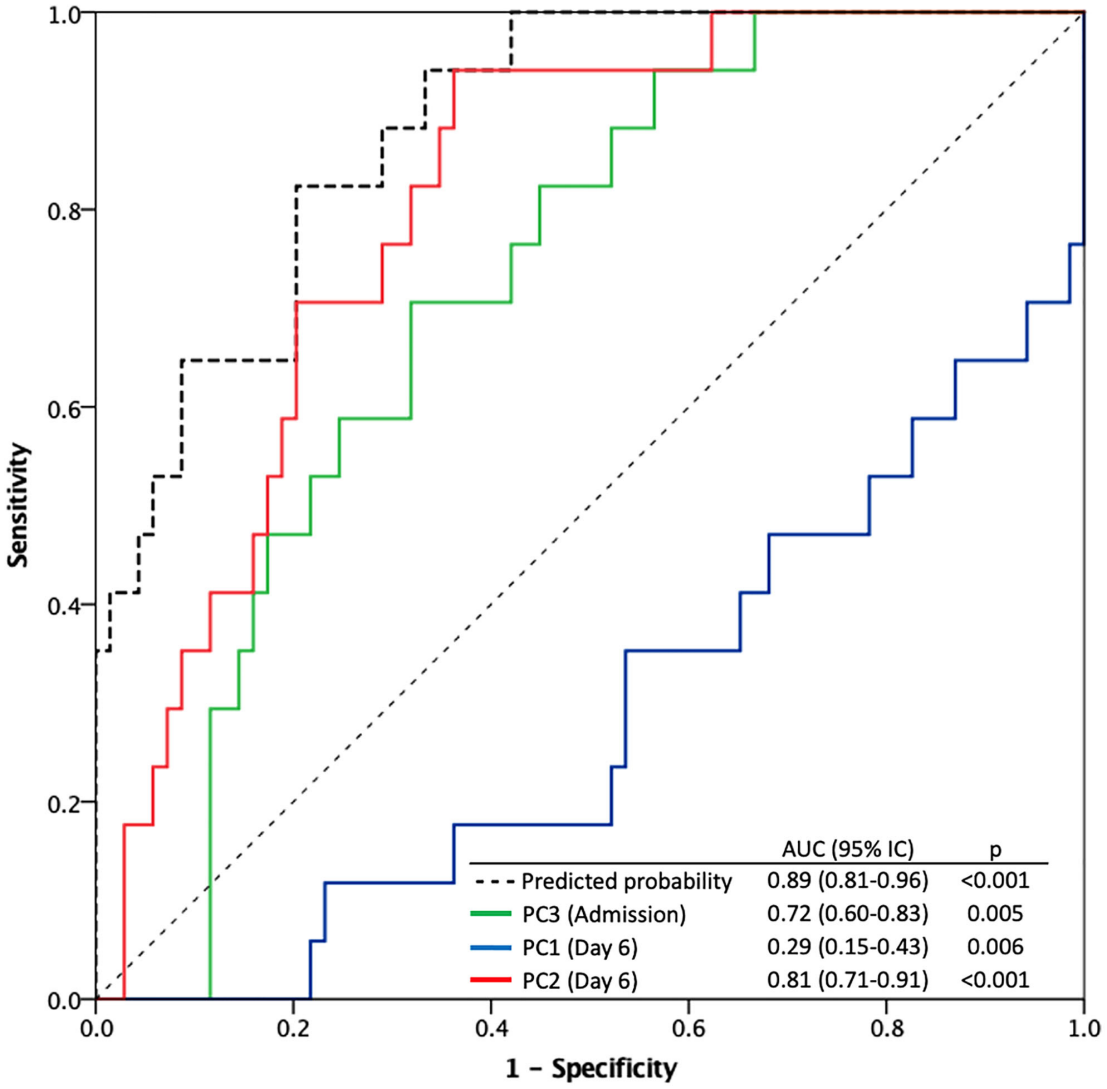
Receiver operating characteristic (ROC) curves for the predicted probability of the logistic regression model and significant predictors for 28-day mortality (PC3 at the day of admission, PC1 and PC2 at the sixth day after admission).

Sex and age between both groups were not significant variables in the final adjusted model. Logistic regression analysis (**Table S5**) showed that on the day of admission, only the combination of cytokines defined by PC3 was significant [OR: 2.35, 95%CI: 1.09-5.06, p=0.029]. In contrast, PC1 and PC2 were significant on the sixth day of admission, where PC1 cytokines represented a protective factor relative to mortality at 28 days [OR: 0.273, 95%CI: 0.12-0.64, p=0.003] while PC2 was pointed out as the major risk factor [OR: 5.24, 95%CI: 2.03-13.48, p=0.001]. ROC curves of significant factors and predicted probability for final logistic regression model are shown in **Figure 3**. The area under the curve (AUC= 0.89, 95%CI= 0.81-0.96, p <0.001) shows a good discrimination ability of the model.

## Discussion

Several studies have been carried out comparing cytokine profiles in severe and moderate COVID-19 (20–23). However, there are fewer studies comparing differences in cytokine profiles between survivors and non-survivors or analyzing the relationship between the evolution of cytokines profile in COVID-19 hospitalized patients and mortality (17, 24). Severe disease is characterized by acute lung injury (ALI) that can eventually lead to highly lethal acute respiratory distress syndrome (ARDS) and a cytokine storm (25, 26). Previous studies have associated the significant elevation of many cytokines such as IL-2, IL-7, IL-10, GSCF, IP-10, MCP-1, MIP-1a and TNF-α in the blood of patients with severity of the disease. Particularly the level of IL-6 and IL-10 (27–30). However, we found three cytokine clusters (PC1, PC2 and PC3) that could be considered as independent risk factors. Their different contribution would be associated to a higher or lower mortality risk. On one hand, IL-2, IL-15 and BDNF levels (PC1) are significantly reduced in the group of non-survivors, which suggests they constitute a protective factor. In contrast, cytokines included in PC2 (HGF, SCF, IL-18, MCP-1 and eotaxin) together with the combination of PC3 (IL-1α and VEGFA) are significantly increased in non-survivors representing a risk factor for mortality at 28 days. It is interesting that, while PC1 and PC2 are significantly reduced and increased, respectively, at the three stages evaluated; PC3 only showed to be significantly increased in non-survivors at the day of admission. In addition, whereas no trend is observed in PC1 or PC3, PC2 seems to display an increasing trend through time.

PC3, constituted by the combination of IL-1α and VEGFA is only significantly increased at day of admission. The importance of this set at day 0 may be explained by its implication in the initial response to COVID-19 severe infection. Initial COVID-19 studies revealed higher levels of IL-1α, which is a cytokine of initial states of the innate immune response (31). Actually, IL-1α has recently been described as an early marker to predict a bad outcome in COVID-19 severe patients (23). This could be explained by the fact that the precursor of IL-1α, in contrast to IL-1β or IL-1ra, does not need to be activated and is capable of triggering a powerful inflammatory response when it is released by damaged cells (32). Its main biological activities are activation of T lymphocytes, and B cell proliferation along with synthesis of immunoglobulins (31). On the other hand, VEGFA has a key role in lung development resulting in an appropriate organization of the pulmonary vascular network (33). VEGFA can be released during hypoxia, in inflammatory situations where there is damage to the endothelium. Its effect is mediated by VEGFR-1 and VEFGR-2 that contribute to hematopoiesis and monocyte chemotaxis and increased permeability, respectively. PC3 component could be interpreted as the initial expression of the existence of a severe cell damage in lungs due to macrophage infiltration produced by SARS-CoV-2 virus (34), which would initiate the subsequent fatal pathogenesis and exacerbate the immune response observed in following stages of disease. As a matter of fact, our results support previous studies that prove the early use in severe disease of treatments with IL-1 antagonists such as anakinra, improves outcome by blocking progression of the cytokine storm (35).

Our results showed that despite cytokine levels were increased in both survivors and non-survivors, in the first group levels remain similar through time. However, there is a significant upward trend in PC2 in non-survivors through time, which could remark their importance as mortality markers, as they are particularly elevated at day 6. The cytokines of PC2 are HGF, SCF, IL-18, MCP-1 and eotaxin. The importance of PC2 is that, altogether, represent the intense and probably irretrievable damage in lung tissue due to the virus itself as well as the extreme immune response. First, IL-18, a cytokine belonging to the IL-1 family and intervening in cellular immune response it is secreted upon macrophage activation in viral infections and courses with endothelial damage in lung tissue (32). IL-18 binds in its mature form to specific receptor IL-1 receptor 5 (IL-1R5, known as IL-18 receptor alpha chain), leading to the recruitment of the coreceptor, IL-1 receptor 7 (IL-1R7, known as IL-18 receptor beta chain). IL-18BP, a natural inhibitor of IL-18, circulates maintaining a balance between both of them. Severe COVID-19 patients have been shown to have an imbalance of IL-18/IL-18BP (36, 37). Actually, an antibody against IL-1R7 has been proposed to reduce inflammatory signalling of IL-18 in COVID-19 patients (38). SCF, HGF and eotaxin are upregulated by inflammatory processes and secreted by mast cells upon cytokine stimulation including IL-18. All of them have also been related to severe lung injury. Eotaxin and SCF play a central role in mast cell and eosinophilic infiltration and, SCF promotes the expression and release of MCP-1 from lung mast cells (39–41). In addition, HGF can promote the development of different cell lineages, including thrombocytes, as to contribute to tissue repair and modulate the adaptive immune response to control inflammation (15, 42). Worthy of remark is that HGF and eotaxin were found to be elevated in patients with severe influenza A (H1N1) virus and other viral infections and in patients with inflammatory lung injury (43–46).. Finally, we have MCP-1, which has been previously described as a factor to predict severity in COVID-19 patients due to its activity in monocyte recruitment to arterial wall (47). More than 70% of the deaths related to COVID‐19 are associated with deregulation of the mechanisms that control blood clotting, as a part of the innate immune response to limit pathogen spread in a process known as immunothrombosis (48, 49). Moreover, hypoxic environment due to pulmonary affection activates pro‐coagulation factors that may promote thrombosis (50). In our work, both MCP-1 and D-dimer are elevated in non-survivors indicating thrombotic events were taking place. Taking together PC2, has a potential as a marker of bad prognosis due to extreme inflammation and lung injury.

According to the logistic regression model, the most contributing factor to survival in this case is a protective factor for mortality that involves high plasma levels of the cytokines IL-2, IL-15 and BDNF in survivors compared to those determined in deceased patients. The elevation of the plasmatic levels of some of these three cytokines would, therefore, increase this protective factor. BNDF has a pivotal role in neuroplasticity that is considerably affected by inflammatory states. The most common pro-inflammatory cytokines are IL-1β, IL-6, TNF-α and IFN-γ cause a significant reduction of BDNF gene expression (51). In fact, low serum BDNF levels were correlated with severe SARS-CoV-2 infection in a recent study where BDNF levels were restored during patients´ recovery. This has been linked to the lymphopenia observed in critical COVID-19 patients as lymphocytes contribute to peripheral BNDF secretion (52). IL-2 and IL-15 have several similar functions. Both cytokines stimulate the proliferation of T cells; induce the generation of cytotoxic T lymphocytes; facilitate the proliferation of B cells as well as immunoglobulin secretion; and induce generation and persistence of natural killer cells (53). On one hand, IL-15 has a pivotal role in viral clearance by long-lasting, high avidity T-cell responses to invading pathogens by ensuring survival of memory cells. Actually, influenza and other respiratory viruses induce peripheral and local expression of IL-15, which is critical for anti-viral responses by different lymphocyte populations. Low levels of circulating IL-15 have been associated with high viremia and poor disease outcome (54). On the other hand, IL-2 is involved in maintenance of peripheral regulatory T cells and elimination of self-reactive ones (53). What is more important, both IL-2 and IL-15 can regulate cell proliferation, thus controlling excessive response (31).The progressive decrease of BDNF, IL-15 and IL-2 in plasma may be a warning factor of disease deterioration in severe patients with COVID-19 pneumonia. For that matter, IL-2 and IL-15 supplementation have been proposed as to improve the immune disorder and reduce mortality (55, 56).

The independence of the three factors suggests three possible independent therapeutic pathways of intervention. One aimed at stopping the initial proinflammatory response by blocking IL-1 and VEGFA. Other aimed at regulating the immune response by increasing IL-15 levels, IL-2 and BDNF. And other aimed at limiting lung tissue by interfering with HGF, SCF, IL-18, MCP-1 or eotaxin. As a multiphasic disease, immune monitoring and multiple targets would be a great approach to improve outcomes.

The main limitations of the study are that we only performed a plasma analysis including 45 cytokines. This means some relevant cytokines in severity of COVID-19, might not be included. However, we selected this panel as it was the most complete available for cytokine analysis and eligible for clinical implementation. As future directions, we considered it would be interesting to confirm our results, especially in relevant cytokines by a classic ELISA analysis. In addition, we consider it would be interesting to evaluate cytokine profiles in healthy population and how these levels correlate with clinical parameters and outcomes. Also, a further multicentric corroboration of our findings would be of interest.

## Conclusions

In summary our findings unravel the cytokine evolution in relation to mortality caused by COVID-19 disease. We propose the use of PC2 associated with clinical data as a marker of strong lung damage, and evolution of values of PC1 and PC3 to predict outcome thus personalize treatment. In addition, we consider early target of the markers of dysregulation of immune responses and therapies to promote tissue repair could be a better approach in severe patients as antiviral therapy could not be useful at that point.

## Data availability statement

The raw data supporting the conclusions of this article will be made available by the authors, without undue reservation.

## Ethics statement

This study was reviewed and approved by Valladolid Hospital’s Clinical Ethics Committee (CEIm) (cod: PI 20-1717). The patients/participants provided their written informed consent to participate in this study.

## Author contributions

All authors contributed to the article and approved the submitted version. EG-S, ET, AT-V and MM-F designed the methodology of the study. AM-G, GQ, RG-B, MP-J and EA-F collected the samples and clinical data. AT-V and LSP supervised the laboratory work. IF-L performed the statistical analysis and prepared the figures with the help of ET and MM-F. LSP and OG-G prepared the original draft. All authors contributed to the article and approved the submitted version.

## Funding

This work was supported by Instituto de Salud Carlos III (COV20/00491, PI18/01238, CIBERINFEC CB21/13/00051), Junta de Castilla y León (VA321P18, GRS 1922/A/19, GRS 2057/A/19), Consejería de Educación de Castilla y León (VA256P20) and Fundación Ramón Areces (CIVP19A5953). LSP received a Río Hortega grant (CM20/00138) from Instituto Carlos III (Co-funded by European Regional Development Fund/European Social Fund “A way to make Europe”/”Investing in your future”).

## Conflict of interest

The authors declare that the research was conducted in the absence of any commercial or financial relationships that could be construed as a potential conflict of interest.

## Publisher’s note

All claims expressed in this article are solely those of the authors and do not necessarily represent those of their affiliated organizations, or those of the publisher, the editors and the reviewers. Any product that may be evaluated in this article, or claim that may be made by its manufacturer, is not guaranteed or endorsed by the publisher.
