# Supplementary material for: Time evolution of cytokine profiles associated with mortality in COVID-19 hospitalized patients
Source: Front Immunol. 2022 Sep 27;13:946730. doi: 10.3389/fimmu.2022.946730 (PMC9551198; doi:10.3389/fimmu.2022.946730)
Supplement: Supplementary file 1 [file Table_1.docx]

Supplementary Material

# Supplementary Tables

**Table S1**: Comparison of the cytokine profile on the first day of hospital admission.

|  | Non-survival at 28 days  (N=20) | Survivors  (N=88) | p-value |
| --- | --- | --- | --- |
| - Citokines [median (IQR)] | | | |
| BDNF | 56.33 (77.63) | 63 (179.78) | 0.462 |
| EGF | 2.16 (10.52) | 2.25 (7.55) | 0.918 |
| Eotaxin | 13.65 (9.98) | 14.28 (9.97) | 0.629 |
| FGF2 | 0.69 (1.86) | 1.03 (2.63) | 0.522 |
| GMCSF | 9.53 (7.56) | 12.86 (31.34) | 0.110 |
| GROa | 2.48 (4.19) | 3.25 (4.24) | 0.393 |
| HGF | 269.75 (636.25) | 160.25 (208.48) | 0.020 |
| IFNa | 0.44 (1.83) | 0.51 (1.65) | 0.931 |
| IFNg | 7.63 (11.83) | 8.99 (6.67) | 0.893 |
| IL1a | 7.52 (11.12) | 1.83 (7.44) | 0.040 |
| IL1b | 5.70 (5.85) | 7.01 (10.62) | 0.103 |
| IL10 | 1.4 (2.54) | 1.79 (2.56) | 0.253 |
| IL12p70 | 3.10 (2.43) | 3.71 (3.21051825) | 0.443 |
| IL13 | 1.59 (1.55) | 2.33 (3.1) | 0.296 |
| IL15 | 7.12 (17.09) | 13.83 (19.10) | 0.033 |
| IL17a | 3.87 (10.08) | 7.36 (15.68) | 0.054 |
| IL18 | 58.98 (57.68) | 45.58 (50.74) | 0.319 |
| IL1RA | 627.42 (1358.75) | 604.75 (992.85) | 0.906 |
| IL2 | 8.02 (10.85) | 15.3 (21.01) | 0.037 |
| IL21 | 1.69 (6.18) | 4.18 (13.04) | 0.091 |
| IL22 | 4.66 (46.68) | 3.65 (20.56) | 0.788 |
| IL23 | 6.20 (6.64) | 7.89 (9.84) | 0.279 |
| IL27 | 11.78 (20.91) | 20.62 (38.20) | 0.380 |
| IL31 | 4.25 (6.3) | 5.51 (8.45) | 0.347 |
| IL4 | 5.07 (4.85) | 5.84 (7.43) | 0.504 |
| IL5 | 3.55 (6.10) | 7.39 (20.88) | 0.179 |
| IL6 | 11.64 (30.23) | 13.07 (21.79) | 0.382 |
| IL7 | 1.13 (2.64) | 1.81 (3.03) | 0.522 |
| IL8 | 1.45 (4.66) | 2.14 (3.57) | 0.566 |
| IL9 | 1.59 (2.23) | 2.29 (3.97) | 0.187 |
| IP1b | 43.68 (34.18) | 49.78 (46.85) | 0.506 |
| IP10 | 48.78 (53.21) | 45.4 (42.51) | 0.710 |
| LIF | 11.26 (12.89) | 16.15 (16.84) | 0.329 |
| MCP1 | 41.75 (71.28) | 37.88 (30.85) | 0.226 |
| MIP1a | 3.35 (11.01) | 3.39 (11.96) | 0.443 |
| NGFb | 3.61 (2.72) | 4.37 (3.47) | 0.275 |
| PDGFBB | 265 (564) | 334.92 (718.3) | 0.740 |
| PIGF1 | 25.35 (162.61) | 5.45 (49.81) | 0.467 |
| RANTES | 23.43 (21.09) | 22.15 (17.66) | 0.731 |
| SCF | 9.06 (9.55) | 6.46 (6.8) | 0.319 |
| SDF1a | 649.25 (927.63) | 669.5 (603) | 0.943 |
| TNFa | 6.14 (7.579621) | 6.13 (11.42) | 0.424 |
| TNFb | 2.48 (2.93) | 3.41 (4.92) | 0.176 |
| VEGFA | 262.25 (277.38) | 113.25 (164.49) | 0.005 |
| VEGFD | 6.72 (18.10) | 12.75 (12.86) | 0.192 |

Continuous variables are represented as [median, (interquartile range, IQR)].

**Table S2**: Comparison of the cytokine profile on the third day of hospital admission.

|  | Non-survival at 28 days  (N=20) | Survivors  (N=88) | p-value |
| --- | --- | --- | --- |
| - Citokines [median (IQR)] | | | |
| BDNF | 36.46 (54.84) | 63.4 (201.05) | 0.029 |
| EGF | 1.41 (2.24) | 1.88 (4.25) | 0.326 |
| Eotaxin | 9.78 (12.60) | 14.2 (10.08) | 0.046 |
| FGF2 | 0.67 (1.29) | 0.92 (2.21) | 0.237 |
| GMCSF | 11.95 (16.28) | 12.96 (28.17) | 0.612 |
| GROa | 2.78 (6.15) | 2.39 (2.25) | 0.462 |
| HGF | 573.75 (616.5) | 212 (286.5) | 0.001 |
| IFNa | 0.20 (0.38) | 0.29 (0.50) | 0.322 |
| IFNg | 9.33 (12.46) | 9.04 (5.82) | 0.487 |
| IL1a | 2.96 (6.22) | 2.27 (6.78) | 0.804 |
| IL1b | 5.24 (5.50) | 7.28 (9.1) | 0.079 |
| IL10 | 1.36 (2.57) | 1.41 (1.33) | 0.930 |
| IL12p70 | 3.08 (1.82) | 3.76 (2.98) | 0.150 |
| IL13 | 2.04 (2.11) | 2.41 (3.29) | 0.761 |
| IL15 | 8.85 (11.65) | 15.55 (13.63) | 0.011 |
| IL17a | 5.15 (6.75) | 7.61 (12.13) | 0.063 |
| IL18 | 66.35 (106.95) | 41.8 (56.3) | 0.030 |
| IL1RA | 552.25 (1501.86) | 496 (951.5) | 0.707 |
| IL2 | 12.15 (13.2) | 15.7 (17.66) | 0.086 |
| IL21 | 2.06 (5.73) | 3.66 (11.66) | 0.174 |
| IL22 | 1.62 (20.56) | 3.77 (14.17) | 0.592 |
| IL23 | 7.28 (8.06) | 7.26 (8.16) | 0.655 |
| IL27 | 16.4 (24.47) | 20.48 (48.56) | 0.338 |
| IL31 | 4.56 (5.13) | 5.08 (7) | 0.587 |
| IL4 | 4.64 (4) | 5.55 (5.84) | 0.288 |
| IL5 | 4.20 (9.90) | 5.07 (21.07) | 0.523 |
| IL6 | 6.66 (33.77) | 9.16 (18.39) | 0.879 |
| IL7 | 1.74 (1.9) | 1.8 (2.36) | 0.952 |
| IL8 | 1.2 (1.85) | 2.07 (5.59) | 0.119 |
| IL9 | 1.79 (2.3) | 2.10 (3.12) | 0.322 |
| IP1b | 44.08 (46.63) | 50.4 (47.7) | 0.448 |
| IP10 | 29.5 (53.14) | 24.75 (32.05) | 0.265 |
| LIF | 9.92 (17.33) | 13.35 (13.62) | 0.592 |
| MCP1 | 34.78 (37.54) | 31.9 (33.55) | 0.149 |
| MIP1a | 2.52 (10.44) | 3.21 (9.08) | 0.615 |
| NGFb | 3.88 (3.03) | 4.22 (3.40) | 0.930 |
| PDGFBB | 562 (752.85) | 409.75 (1153) | 0.777 |
| PIGF1 | 21.51 (77.97) | 4.76 (61.51) | 0.968 |
| RANTES | 25.98 (34.07) | 27.15 (23.6) | 0.857 |
| SCF | 11.24 (22.20) | 7.155 (5.44) | 0.360 |
| SDF1a | 579.25 (960) | 724.5 (651) | 0.584 |
| TNFa | 5.73 (11.38) | 7.07 (8.97) | 0.549 |
| TNFb | 2.76 (3) | 3.34 (3.96) | 0.314 |
| VEGFA | 173.37 (375.5) | 101.3 (160.85) | 0.072 |
| VEGFD | 7.27 (9.22) | 11.3 (11.18) | 0.065 |

Continuous variables are represented as [median, (interquartile range, IQR)]

**Table S3**: Comparison of the cytokine profile on the sixth day of hospital admission.

|  | Non-survival at 28 days  (N=20) | Survivors  (N=88) | p-value |
| --- | --- | --- | --- |
| - Citokines [median (IQR)] | | | |
| BDNF | 33.45 (65.08) | 49.75 (122.38) | 0.094 |
| EGF | 1.49 (2.41) | 1.766 (5.044) | 0.661 |
| Eotaxin | 16.9 (11.53) | 14.1 (10.48) | 0.645 |
| FGF2 | 0.58 (0.92) | 0.78 (1.15) | 0.451 |
| GMCSF | 8.18 (22.89) | 11.94 (33.29) | 0.157 |
| GROa | 1.8 (1.54) | 2.21 (1.82) | 0.491 |
| HGF | 1022 (1398.75) | 265 (359.75) | 0.000 |
| IFNa | 0.21 (0.37) | 0.24 (0.31) | 0.833 |
| IFNg | 7.73 (10.49) | 8.87 (5.78) | 0.782 |
| IL1a | 2.76 (14.11) | 1.72 (6.35) | 0.377 |
| IL1b | 4.51 (9.25) | 6.5 (7.41) | 0.090 |
| IL10 | 1.28 (2.11) | 1.42 (1.17) | 0.961 |
| IL12p70 | 2.97 (1.18) | 3.53 (2.28) | 0.157 |
| IL13 | 1.65 (1.99) | 2.08 (2.8) | 0.199 |
| IL15 | 8.51 (14.84) | 14.8 (16.42) | 0.035 |
| IL17a | 4.65 (8.42) | 6.59 (11.07) | 0.117 |
| IL18 | 74.1 (60.48) | 38.25 (48.9) | 0.010 |
| IL1RA | 316 (1223.35) | 473 (1072.49) | 0.786 |
| IL2 | 8.32 (11.31) | 14.75 (21.77) | 0.064 |
| IL21 | 1.93 (5.6) | 3.2 (7.7) | 0.547 |
| IL22 | 1.59 (15.97) | 3.23 (11.63) | 0.961 |
| IL23 | 5.59 (4.55) | 7.043 (6.31) | 0.321 |
| IL27 | 11.54 (24.65) | 16.31 (45.45) | 0.316 |
| IL31 | 3.79 (3.56) | 4.88 (4.53) | 0.326 |
| IL4 | 4.22 (2.15) | 5.37 (4.94) | 0.151 |
| IL5 | 3.05 (4.47) | 3.64 (8.45) | 0.458 |
| IL6 | 12.85 (28.88) | 9.01 (19.18) | 0.909 |
| IL7 | 1.144 (2.30) | 1.64 (2.52) | 0.180 |
| IL8 | 2.3 (11.46) | 2.12 (9.85) | 0.692 |
| IL9 | 1.61 (1.68) | 1.99 (2.12) | 0.471 |
| IP1b | 39.75 (49.93) | 39.85 (46.63) | 0.645 |
| IP10 | 16.8 (21.58) | 17.3 (14.05) | 0.745 |
| LIF | 10.39 (22.01) | 10.47 (14.1) | 0.676 |
| MCP1 | 49.5 (29.13) | 24.6 (34.38) | 0.027 |
| MIP1a | 2.26 (8.26) | 2.72 (8.22) | 0.522 |
| NGFb | 3.65 (1.28) | 3.85 (2.38) | 0.505 |
| PDGFBB | 453.5 (881.45) | 401.5 (1043.5) | 0.633 |
| PIGF1 | 59.05 (102.22) | 7.5 (64.49) | 0.395 |
| RANTES | 27.85 (17.73) | 30.15 (19.3) | 0.892 |
| SCF | 16.15 (21.03) | 6.16 (7.33) | 0.017 |
| SDF1a | 917.5 (1309.5) | 571 (544) | 0.125 |
| TNFa | 6.78 (8.96) | 6.19 (9.90) | 0.733 |
| TNFb | 2.52 (2.2) | 3.02 (2.81) | 0.445 |
| VEGFA | 250 (293.1) | 82.15 (146.28) | 0.017 |
| VEGFD | 9.59 (5.30) | 12.47 (9.88) | 0.170 |

Continuous variables are represented as [median, (interquartile range, IQR)]

**Table S4:** KMO and Bartlett's Test

| Kaiser-Meyer-Olkin Measure of Sampling Adequacy. | | 0.697 |
| --- | --- | --- |
| Bartlett's Test of Sphericity | Approx. Chi-Square | 1066.383 |
|  | df | 45 |
|  | Sig. | 0.000 |

Sig. Significance.

**Table S5:** forward logistic regression of principal components for all sampling times.

|  | B | Std. Error | Wald | *P-value* | OR | CI 95% | |
| --- | --- | --- | --- | --- | --- | --- | --- |
|  |  |  |  |  |  | **Low** | **High** |
| PC3 day 0 | -2.735 | .600 | 20.789 | 0.029 | 2.35 | 1.09 | 5.06 |
| PC1 day 6 | .855 | .391 | 4.790 | 0.003 | 0.273 | 0.12 | 0.64 |
| PC2 day 6 | -1.298 | .436 | 8.853 | 0.001 | 5.24 | 2.03 | 13.48 |

CI, confidence interval; OR, Odds ratio
